# Severe re-expansion pulmonary edema after chest tube insertion for the treatment of spontaneous pneumothorax: A case report

**DOI:** 10.1097/MD.0000000000028259

**Published:** 2021-12-17

**Authors:** In-Hag Song

**Affiliations:** Department of Thoracic and Cardiovascular Surgery, Soonchunhyang University Cheonan Hospital, Chenan-si, Chungcheongnam-do, South Korea.

**Keywords:** chest tube insertion, life-threatening condition, pneumothorax, re-expansion pulmonary edema

## Abstract

**Rationale:**

Re-expansion pulmonary edema (REPE) is a rare complication after chest tube insertion for the treatment of spontaneous pneumothorax. However, this complication can be life threatening when it occurs. Therefore, it is necessary to recognize REPE early and treat it appropriately. In the present study, we report a severe REPE case occurring after chest tube insertion in a patient with spontaneous pneumothorax.

**Patient concerns:**

A 27-year-old male patient visited out hospital with chest pain on the left, which had started a week ago. After diagnosed with pneumothorax and having chest tube insertion, the patient complained of sudden shortness of breath, persistent cough, foamy sputum, and vomiting.

**Diagnosis:**

Based on the symptoms and imaging findings, the patient was diagnosed as REPE.

**Interventions:**

After the condition of the patient deteriorated rapidly, he was transferred to intensive care unit and then mechanical ventilation and conservative treatment were performed after endotracheal intubation.

**Outcomes:**

After mechanical ventilation and conservative treatment in the intensive care unit, the symptoms and radiological findings improved, and then mechanical ventilation was weaned and the chest tube was removed from the patient. However, due to recurrent pneumothorax after removal of the chest tube, video assisted thoracoscopic surgery (VATS) wedge resection was performed. At 6 months post-operative follow up, he was well with normal radiological findings.

**Lessons:**

REPE occurs rarely, but once it does, it causes a serious condition that can be life-threatening. Therefore, patients with the risk factors related to it should receive a closed observation after chest tube insertion. Moreover, if REPE occurs, appropriate treatments should be carried out by recognizing it early.

## 1 Introduction

Re-expansion pulmonary edema (REPE) occurs when a collapsed lung rapidly re-expands for a short time after chest tube insertion to treat pneumothorax, pleural effusion, or hemothorax, but its exact pathophysiology is unclear.^[1–3]^ REPE is a rare complication with an incidence of <1% in pneumothorax, but a mortality rate of up to 20%, and so it is very fatal.^[3–5]^ Therefore, it is necessary to recognize the occurrence of REPE early and perform appropriate treatments. In this study, we report on a serious REPE case that occurred after chest tube insertion in a patient whose left lung was totally collapsed due to pneumothorax.

## 2 Case report

**Figure 1:**
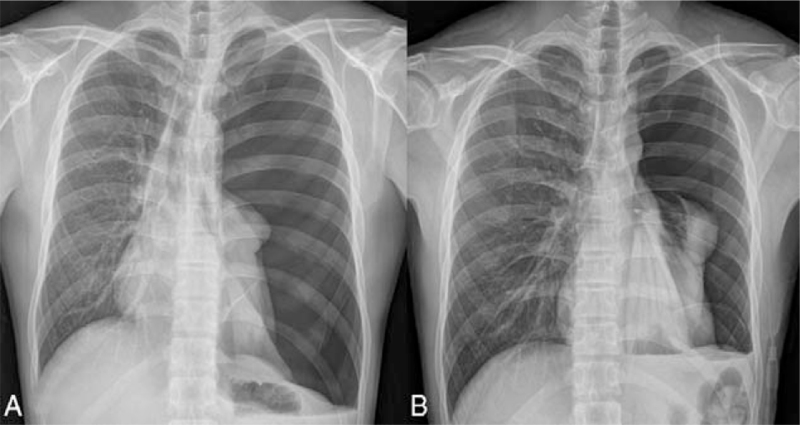
Chest x-ray at the time of admission show left sided complete pneumothorax with total lung collapse (A). A follow up chest x-ray at the day after chest tube insertion show the left lung is not expanded much (B).

**Figure 2:**
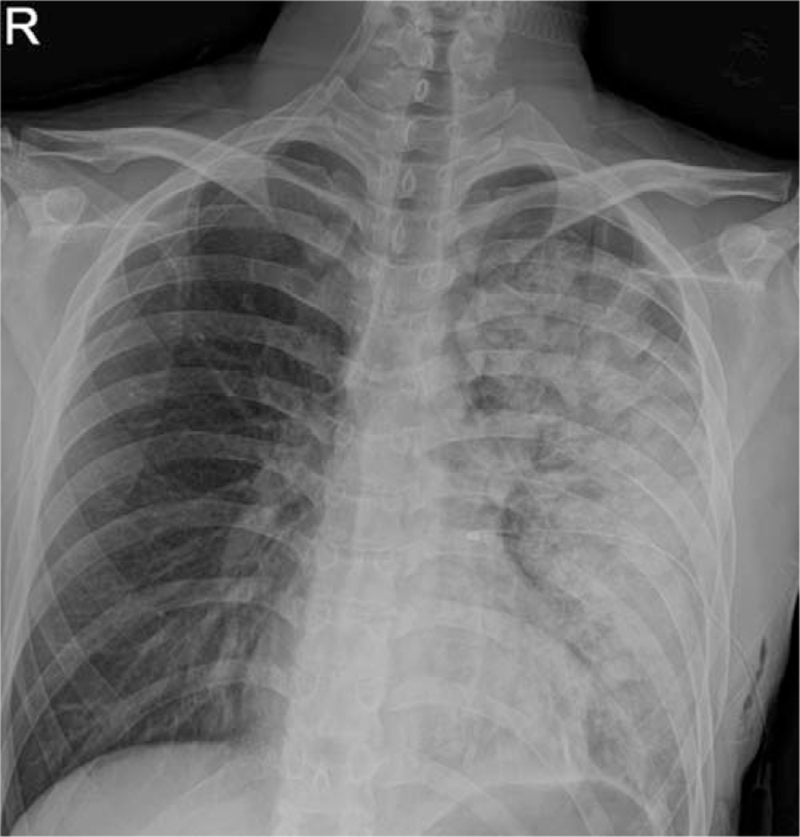
A chest x-ray followed up after the onset of symptoms such as severe breathing difficulties and cough with foamy sputum. A newly developed ill-defined consolidation is observed in the left lung.

**Figure 3:**
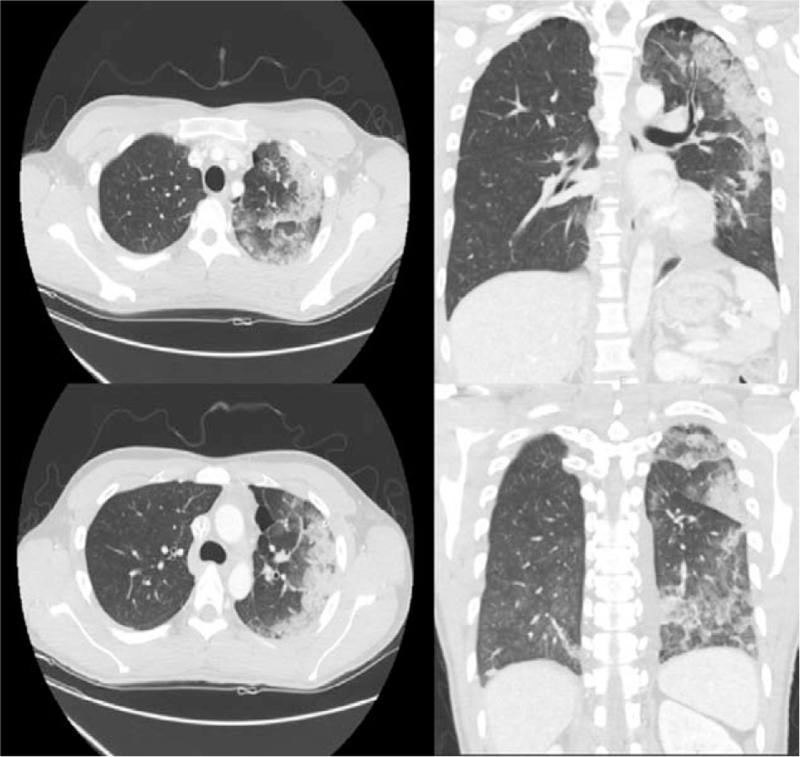
Chest computed tomography (CT) scan shows multiple irregular patchy consolidation and interlobular septal thickening in the left lung.

**Figure 4:**
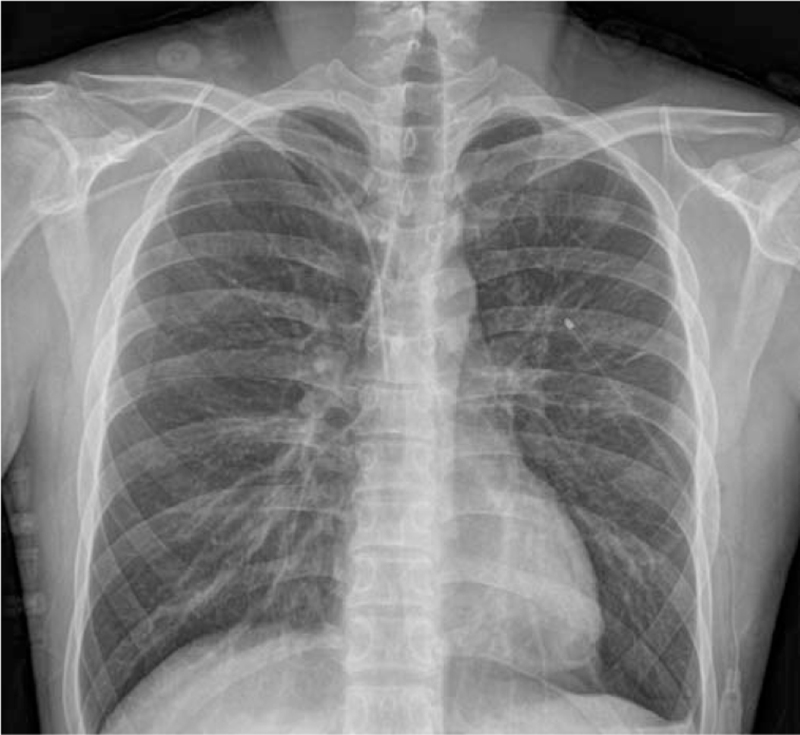
Chest x-ray taken at 6 days after the onset of re-expansion pulmonary edema. The pulmonary edema of the left lung improved.

A 27-year-old male patient was admitted to the emergency room of this hospital with shortness of breath that started a week before admission. A chest x-ray performed at emergency room showed a left-sided pneumothorax (Fig. 1A), and the patient was referred to the department of thoracic surgery. The left lung of the patient was totally collapsed, and there was a high possibility of REPE upon chest tube insertion with the symptom onset of a week. Accordingly, after explaining this situation to the patient and his caregivers, a 12 French trocar tube was inserted into the left thoracic cavity of the patient. In order to allow the lungs to expand slowly, natural drainage was performed without suction, and the rubber tube connecting the chest tube and the water seal bottle was partial clamped. On the day after chest tube insertion, there was an finding of air leakage through the chest tube, but the lungs were not found to expand much as a result of chest x-ray (Fig. 1B), thus the partial clamped rubber tube was de-clamped. However, about an hour after de-clamping, the patient suddenly complained of severe respiratory distress, cough with foamy sputum, and vomiting. Immediately the chest tube was clamped to stop drainage. With oxygen saturation measured 75%, oxygen was administered to the patient via an oxygen mask, but no improvement was observed. In turn, a chest x-ray was followed during additional high flow nasal prong (HFNP) therapy, exhibiting REPE findings such as newly developed ill-defined consolidation in the left lung (Fig. 2). Oxygen saturation did not improve even after HFNP therapy, and further, persistent cough with foamy sputum, shortness of breath, and a drop in blood pressure occurred. With the need for intensive care recognized, the patient was immediately transferred to the intensive care unit and then was sedated and intubated to perform mechanical ventilation. After supportive care including mechanical ventilation, the condition of the patient was improved and then extubation was performed two days later. On the third day, chest computed tomography (CT) (Fig. 3) was performed, and the patient was transferred to a general ward. From the 6th day, a chest x-ray showed no more edema (Fig. 4). There was no finding of air leakage through the chest tube and a chest x-ray showed full lung expansion, in turn, the chest tube was removed on the 9th day of hospitalization. However, a chest x-ray showed recurrence of pneumothorax the day after removal of the chest tube, and the chest tube was inserted again. Both the patient and his caregivers wanted operation, so the video assisted thoracoscopic surgery (VATS) wedge resection was performed. Following operation, the patient recovered without any complications. For the follow-up at 6 months after operation, no adverse symptoms were found and the imaging findings were also found to be normal.

This case report was approved by the Institutional Review Board of Soonchunhyang University Hospital (SCHCA 2021–09-007). The Patient has provided informed consent for publication of this case.

## 3 Discussion

REPE occurs when a collapsed lung suddenly re-expands after chest tube insertion to treat pneumothorax, large amounts of pleural effusion, or hemothorax. The incidence of REPE, including all etiologies, is 0.9% to 20%, but the majority occurs when the pleural effusion is rapidly drained, and upon pneumothorax, the incidence is <1%.^[3,5–8]^ The pathophysiology of REPE is still unclear. However, increased permeability of damaged pulmonary blood vessels due to rapid re-expansion of lung tissue, histological changes in the lung parenchyma, and reperfusion of ischemic lungs after re-expansion increased oxygen free radicals and anoxic stress, thereby resulting in damage to the vascular endothelium. These damages have been suggested as the onset the mechanism of REPE.^[2,4,6]^

Risk factors of REPE include young age (<40 years old), large pneumothorax (>30%) or large amount pleural effusion, long duration of symptoms and lung collapse (>3 days), rapid re-expansion of the lungs (<10 min), and the pleural effusion drainage of 1.5 L or more at once and negative pressure suction drainage.^[1,4,6,9]^ The patient in this case had risk factors such as age (27 years old), total collapse, and long duration of symptoms (7 days). Therefore, it is important to accurately listen to the patient's medical history, including the period of onset of symptoms, and if a patient has these risk factors, efforts should be provided to expand the lungs slowly. Several authors recommend that no more than 1 L of air or fluid be drained at a time, and that a water valve device should be used instead of suction.^[2,4,10–12]^ The maximum amount of drainage at once should not exceed 1200 to 1800 mL.^[4]^ However, pneumothorax, unlike pleural effusion, is difficult to drain them progressively, and the intermittent clamping of a chest tube may cause subcutaneous emphysema.^[6,13]^ Thus, some authors suggest that, if there are such risk factors, inserting a chest tube after repeatedly aspirating less than 1000 cc of air with a syringe may be a safe and practical solution.^[10]^ We inserted a 12 French trocar tube with a relatively small diameter to slowly expand the lungs of the patient, and partial clamped the rubber tube connecting the chest tube and the water seal bottle. However, de-clamping was performed because the patient's lungs were not expanded well at the day after. Although a suction device was not connected, REPE occurred at about an hour after de-clamping.

Rapid dyspnea and tachypnea, usually occurring within 1 to 2 hours of initiating drainage through a chest tube, are characteristic symptoms of REPE. Chest pain, cough with foamy sputum, tachycardia, hypotension, hypoxia, cyanosis, and nausea and vomiting are also included in those of REPE.^[2,4,9]^ Some authors recommend stopping drainage immediately if a patient starts coughing during drainage, since coughing may be the first symptom of edema development.^[2,4,14]^ This patient had severe dyspnea, cough with foamy sputum, hypotension, hypoxia, and vomiting, thus drainage was stopped immediately.

REPE is usually diagnosed based on a series of characteristic imaging findings along with typical clinical progress.^[6]^ A chest x-ray shows different degrees of unilateral alveolar filling patterns within 2 to 4 hours after re-expansion of the lungs. In addition, a chest CT shows ipsilateral consolidation, interlobular septal thickening, ground-glass opacity (GGO), intralobular interstitial thickening, bronchovascualr bundle thickening, and atelectasis.^[4,6]^ Chest x-ray findings worsen up to 48 hours and persist for 4 to 5 days, and edema improved within 5 to 7 days without adverse imaging findings.^[4,14]^ In this case, ill-defined consolidation was found on chest x-ray immediately after symptom onset in the patient. Since the patient's condition was serious right after symptom onset, intubation and ventilator care were carried out immediately. Accordingly, a chest CT could not be implemented immediately after symptom onset, but it was performed after extubation because the patient improved at 3 days following REPE onset, showing multiple irregular patchy consolidate and interlobular septal thickening. In our patient, symptoms and imaging findings began to improve at 2 days after REPE onset, and edema was no longer observed on the chest x-ray at 6 days.

As such, REPE can be caused suddenly, worsen rapidly, and result in sometimes life-threatening conditions. In this regard, it is necessary to prepare in advance in mind that REPE may occur before chest tube insertion in patients with risk factors. Notably, following chest tube insertion, it is necessary to observe carefully whether REPE occurs, and to recognize it early and take appropriate measures as soon as possible when symptoms suspected of REPE appear.^[2,5,15]^

The management of REPE is supportive care. It consists of oxygen or continuous positive air way pressure (CPAP) support, in some cases intubation with mechanical ventilation will be necessary.^[2,4,6]^ Since intrapulmonary shunting can cause hypoxia or hypovolemia, fluid supply or use of inotropes is required. Also, the use of diuretics should be avoided as they may worsen hypovolemia.^[4,6,9]^ The lateral decubitus position allows a patient to bring the affected area upwards, and so to reduce shunting and increase oxygenation. Unilateral ventilation is rarely required.^[4,16]^

In conclusion, REPE after chest tube insertion is rare in patients with pneumothorax, but it causes a serious condition that can be life-threatening. Therefore, it is necessary to be well-acquainted with the risk factors of REPE, and to make efforts to expand the lungs slowly in patients with the risk factors. Furthermore, it is necessary to be well aware of the symptoms suggestive of REPE, and upon the onset of suspicious symptoms during closed observation of those patients, early recognition and active treatments for it are required.

## Acknowledgments

This research was supported by the Soonchunhyang University Research Fund

## Author contributions

**Conceptualization:** In-Hag Song.

**Data curation:** In-Hag Song.

**Formal analysis:** In-Hag Song.

**Investigation:** In-Hag Song.

**Writing – original draft:** In-Hag Song.

**Writing – review & editing:** In-Hag Song.
